# Seasonality and geographical distribution of Kawasaki disease among Black children in the Southeast United States

**DOI:** 10.3389/fped.2023.1203431

**Published:** 2023-06-27

**Authors:** Luz A. Padilla, Adeniyi J. Idigo, Kathryn Maxwell, Yung Lau, Howard W. Wiener, Sadeep Shrestha

**Affiliations:** ^1^ Department of Epidemiology, School of Public Health, University of Alabama at Birmingham, Birmingham, AL, United States; ^2^ Department of Pediatric Cardiology, School of Medicine, University of Alabama at Birmingham and the Pediatric and Congenital Heart Center of Alabama, Children’s of Alabama, Birmingham, AL, United States

**Keywords:** Kawasaki, seasonality, race, incidence, southeast US

## Abstract

**Introduction:**

Kawasaki Disease (KD) is a leading cause of pediatric acquired heart disease in the United States, affecting up to 7,000 children annually. Seasonal variation, an epidemiological characteristic of KD, has previously been reported predominantly among Asian children; however, little is known about the epidemiology and seasonality of KD of Black children within the U.S.

**Methods:**

Electronic medical records were abstracted from 529 hospitalized KD patients admitted to a single tertiary center in Alabama between 2005 and 2019. Medical charts were reviewed to confirm KD diagnosis following American Heart Association criteria. Cases were stratified by the month of diagnosis date to assess seasonality, and statewide distribution of incidence is reported at county level using geographical spatial analysis. Comparisons were performed between Black patients and White patients with KD.

**Results:**

The average number of KD cases per year was 35. Approximately, 60% were males and 44% were White children (*N* = 234), 45% were Black children (*N* = 240) and 11% were other races (*N* = 55). Black children were younger than White children at KD admission (median age 32 vs. 41 months respectively, *p* = 0.02). Overall, the highest rates of cases occurred between January and April. When stratifying by race, cases started to rise in December among White children with the highest rates between February and April with a peak in March. Among Black children cases were high during the winter season (January–April) with a peak in April. Similarly high rates also occurred in June, July and November. There were no differences in geographical distribution of cases by race.

**Conclusion:**

KD incidence among White children in Alabama follows a seasonal cycle similar to other regions in the U.S. However, sustained incidence and additional peaks outside of the usual KD seasonality were seen among Black children with KD. Further studies are needed to investigate differential triggers between races.

## Introduction

Kawasaki disease (KD), an inflammatory vasculitis, is the number one cause of acquired heart disease among children in the U.S. (1). The cause of KD is unknown, but has been hypothesized to be triggered by an infectious agent (i.e., adenovirus, parvovirus, mycoplasma, Staphylococci) (2). The infectious theory also stems from the incident patterns and seasonality displayed by KD cases, with peaks during winter and spring and low occurrence in the fall, similar to that of some of these pathogens (2–5). Environmental exposures such as weather patterns, humidity and stagnant bodies of water have also been associated with incident patterns of KD (3, 4, 6–10).

In addition to possible environmental triggers, there may be host factors that can affect susceptibility to developing KD (2, 11). KD disproportionately affects Asians (33.3 per 100,000 children younger than 5 years) who carry the highest incidence rate of KD for any race, followed by Black children (23.4 per 100,000 children younger than 5 years) (5, 12). Although the seasonality and epidemicity of KD has been previously described, primarily for children of Asian descent, little is known among Black children due the low representation of this racial group in epidemiological studies (4, 7–10). Furthermore, the inflammatory response and outcomes during KD of Black children differ from that of White children (13, 14). Racial and environmental exposure differences associated with developing KD could affect this racial group's seasonality (4, 13, 14). The purpose of this study is to describe the seasonality and distribution of Black children with KD in Alabama, a Southern State in the United States (US).

## Materials and methods

This is a cross-sectional study conducted at the University of Alabama at Birmingham (UAB) using the electronic medical record (EMR) data of children attending Children's of Alabama (COA), a large tertiary hospital center and the only pediatric hospital within the state of Alabama. A query was conducted using a Pediatric Cardiology departmental internal database (PedCard) using ICD-9 (446.1) and ICD-10 (M30.3) codes to identify children who presented with KD symptoms from January 2005 through the end of 2019. During the retrospective chart review, those with duplicate or missing records were removed. Patient records were then screened and excluded if documentation was lacking in order to meet the American Heart Association (AHA) criteria for KD diagnosis (1). Although records were screened to verify KD diagnosis, a cutoff point was set at the end of 2019 due to the start of the COVID-19 pandemic in the U.S. This was to avoid any inclusion of multisystem inflammatory syndrome in children (MIS-C) hospitalizations, which also has overlapping symptoms with KD (15).

Date of hospital admission, race, gender, patient residence zip code and KD symptoms described in the admission and/or discharge KD hospitalization notes were extracted manually and entered into RedCap (16).

### Statistical analysis

Descriptive statistics (frequencies, percentages, medians, interquartile ranges, means and standard deviations) were used to summarize the data for children hospitalized with KD at our center. The cohort was then stratified by Black and White race. Other race groups were excluded from the comparative analysis due to small numbers. Racial differences between Black and White children were compared using *x*^2^ for categorical variables and, student t-test and Wilcoxon Rank-Sum for the continuous data where appropriate. Statistical Analytical Software (SAS) 9.4 (SAS institute, Cary, NC) was used for the analysis and all statistical tests of a two-sided *p*-value of <0.05 were considered significant. To graph KD seasonality multiple time series for the distribution of KD cases were computed using date of hospitalization. The first set of three time series shows the raw case counts by month throughout the study period (2005–2019) for the entire cohort and then for Black and White children, separately. For the second set of time series, the proportion that were hospitalized for KD was calculated for all years collapsed by a specific month. Next a mean for all proportions for that month across the years was calculated with 95% confidence intervals to assess monthly seasonality trends for the overall cohort and for Black and White children separately.

Additionally, a geographical spatial distribution analysis of Black and White cases of KD hospitalizations using ArcGIS 10.2 (Redlands, CA) was conducted. County-level cumulative incident cases over the study period (cases per 100,000 county population) were standardized using age-and race-adjusted county population based on the 2010 census data from the United States Census Bureau (17). Rural and urban areas were also defined based on 2010 US Census Bureau's urban-rural classification and indicated in the map (18, 19). A gradient of color was used to indicate different ranges of cumulative incidence in the state map by county. Counties with fewer than 3 cases total did not have the cumulative cases calculated and thus the counties did not have indicator values. Counties with no reported cases were recorded as blank.

## Results

Of the 602 patients identified in our query, 47 (7.8%) had a missing hospital admission date and 26 (4.3%) did not meet the AHA KD diagnosis criteria (1). Our final cohort to assess seasonality included 529 children who were hospitalized with KD during our 14 year study period. Baseline characteristics for the cohort are presented in Table 1. The average number of KD cases in Alabama per year was 35. Forty five percent (*n* = 240) of the children who presented with KD were Black, 44% (*n* = 234) were White, and 11% were other races (*n* = 55). The median age during KD admission was 35 months (±2.9 years). Black children who were hospitalized with KD were younger than White children (32 vs. 41 median months respectively; *p* = 0.02). The proportion of males was higher (∼60%) among the cohort with no differences between the two races. The most common symptom reported was rash followed by conjunctivitis and oral symptoms. There were no racial differences between the reported AHA symptom criteria at admission for KD.

**Table 1:** Demographic and symptoms at the time of Kawasaki disease hospitalization (2005–2019) by race.

	Total	Black	White	*p*-Value
	*N* = 529	*N* = 240 (45%)	*N* = 234 (44%)	
Age at admission (months)	35 [16–62]	32 [16–55]	41 [19–67]	0.02
Male	319 (60.5)	143 (59.8)	137 (58.8)	0.82
Symptoms				
Conjunctivitis	354 (66.9)	160 (66.7)	164 (70.1)	0.42
Skin rash	372 (70.3)	164 (68.3)	177 (75.6)	0.08
Cervical lymphadenopathy	175 (33.1)	77 (32.1)	84 (35.9)	0.38
Oral changes	332 (62.8)	153 (63.8)	149 (63.7)	0.99
Swollen hand and feet	286 (54.1)	140 (58.3)	122 (52.1)	0.17

Median [IQR]; *N* (%).

**Figure 1:**
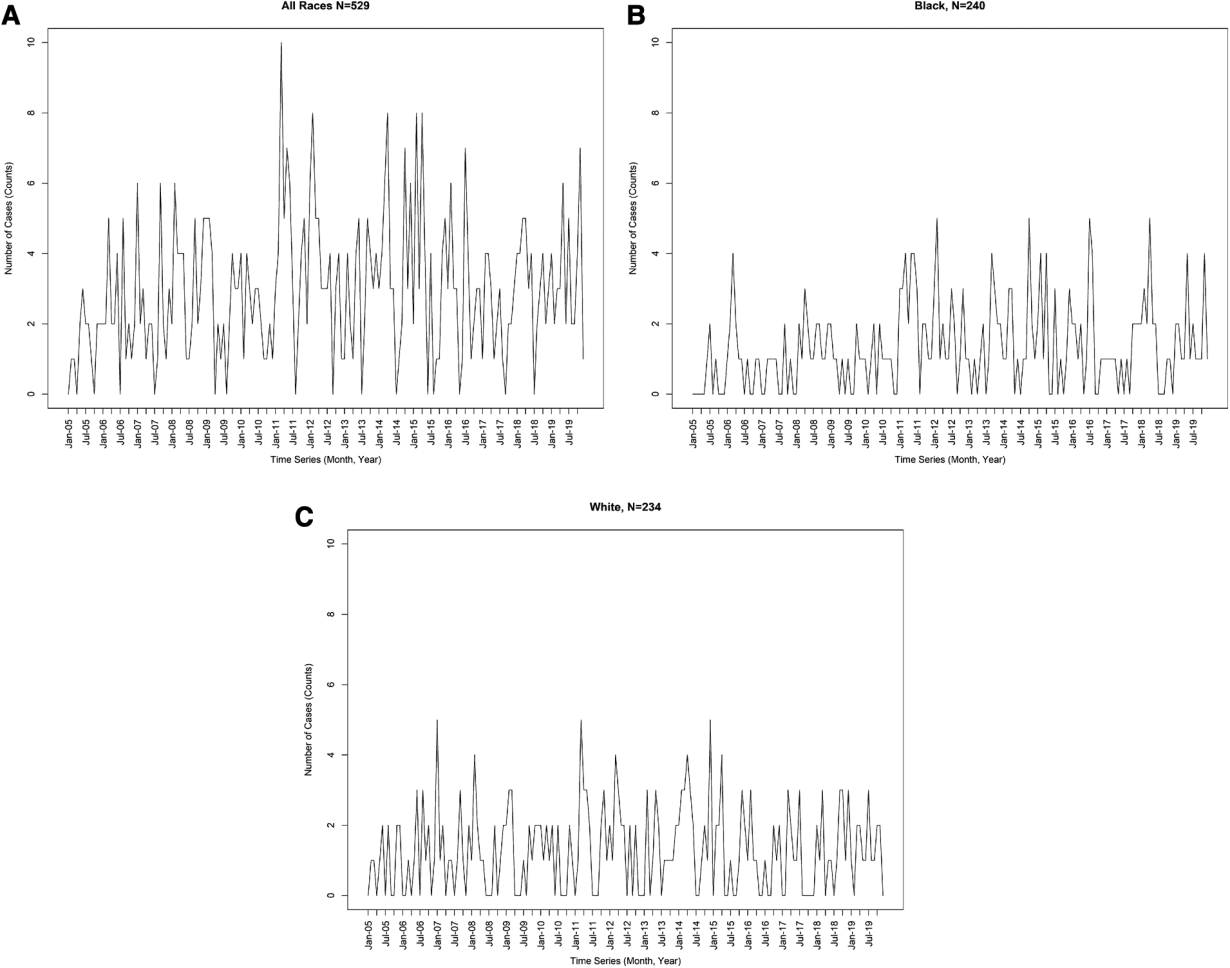
(**A**) Case count for Kawasaki disease hospitalizations from 2005 to 2019. (**B**) Case counts for Black children who were hospitalized with Kawasaki Disease from 2005 to 2019. (**C**) Case counts for White children who were hospitalized with Kawasaki Disease from 2005 to 2019.

Figure 1 shows the raw case count for KD admissions across our study period (2005–2019). In the overall cohort, most months reported 6 cases or fewer with the exception of cases during 2011, 2012, 2014, and 2015 where case counts reached peaks of 8 and 10 cases (Figure 1A). Case counts for Black children in Figure 1B mostly remained low before 2011 (∼2 cases) but started to increase after this year with peaks of 5 in 2012, 2015, 2016 and 2018. Although there was an increase of cases across time, the case count for White children with KD across the years was also low (∼2 cases), with peaks of 5 in 2007, 2011, and 2015 (Figure 1C). All three time series show an upward trend in case counts across time.

**Figure 2:**
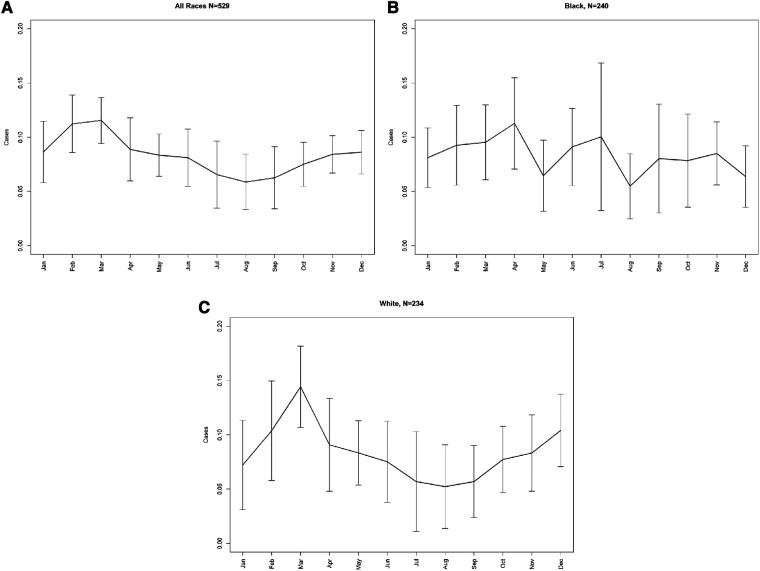
**A** Seasonality for Kawasaki disease hospitalizations by month. (**B**) Seasonality of Kawasaki disease hospitalizations among Black children by month. (**C**) Seasonality of Kawasaki disease hospitalizations among White children by month.

The seasonality for KD hospitalizations by month are shown in Figure 2. In our overall cohort, a rising incidence is seen in January with the highest proportion of cases reported between January and April (∼9%–12% every month) for all cases. The incidence then decreases and is low all summer (June through September, <8%) (Figure 2A). Among Black children, the upward incidence for KD cases occurred during the winter season (January through April) and peaked in April (11.2%). High peaks similar to those observed in the winter were also seen in the summer (June and July, 9%–10%) and fall (November, 9%) although there was a drop of cases in May (6%) (Figure 2B). Among White children, cases start to rise in December and almost half of cases for White children with KD occurred between January and April, with a peak in March (14.4% of cases). The lowest incident rates mimicked the overall cohort (June through September, <8%). August had the lowest incident rates of KD hospitalizations for White children (5.2%) (Figure 2C).

From the initial query, 62 who had no race or missing race, 3 who did not have zipcode and those who did not meet the AHA diagnosis criteria were excluded from the geospatial distribution mapping. When mapping the 256 Black children and 255 White children included in the geographical spatial analysis there was no discernable pattern when comparing KD incident cases between rural and urban counties or the two racial groups (Supplementary Figures S1A,B).

## Discussion

In this study, we assessed the seasonality and distribution of KD among Black and White children in Alabama, during a 14-year period (2005–2019). Alabama poses a unique opportunity to describe the seasonality and epidemicity of KD in the U.S., given it's unique geography (a large portion of its surface area are bodies of water), climate (high humidity) and population distribution (a relatively high proportion of Black individuals, approximately 27%) (20–22). Both proximity to bodies of water and a humid climate are thought to be associated with KD seasonality (3, 4, 6). To our knowledge this is one of the first studies to describe the seasonality of KD among Black children using the largest known cohort of Black children with KD. Our study findings suggest that: (1) seasonality of hospitalizations among Black children with KD remained somewhat steady throughout the year with peaks outside of the traditionally expected seasonality for KD in the U.S.; (2) Black patients were hospitalized with KD at a younger age; and (3) the proportion of KD patients who were hospitalized and are Black (45%) is higher than the proportion of Black children in the State (28%) (23). These findings hold important implications as they add to possible increased susceptibilities and possible differences [i.e., low intravenous immunoglobulin (IVIG) response, higher coronary artery incidence, etc.] faced by Black children who develop KD when compared to other racial groups in the U.S. (12–14).

Previously, seasonality studies of KD in the U.S. have shown an increase in cases during the winter, peaking in early spring, after which cases drop and remain steady for the rest of the year until the next winter season (3, 4, 9, 24–31). The seasonality observed among Black children hospitalized with KD displayed a different pattern in our study. Cases among Black children also had rising rates during the winter and early spring but differed from the typical seasonality in that high rates similar to those occurring in winter were also seen during summer and fall months.

Both of our racial groups were exposed to the similar climates and environments yet Black children still display higher incidence, suggesting potential host susceptibilities. It is hypothesized that both infection and inflammation are involved in KD development. Black children with KD have higher levels of inflammation during their KD (C-Reactive Protein and Erythrocyte Sedimentation Rate) which are thought to predispose them to poor clinical outcomes (poor IVIG response and higher coronary artery aneurysms) (12–14). Although race is a social construct and should not be used as a surrogate for genetics, there is evidence that suggest that individuals with African ancestry have stronger inflammatory responses (33–35). Furthermore, Black children may have predisposition that may increase their risk for KD susceptibility, poor treatment response and sequelae (2, 14, 32, 33, 35). The assumption of both racial groups being exposed to similar environments should be taken with caution as there could be myriad of environmental, cultural, dietary and genetic factors that differ between races that could influence the different findings and were not accounted for in this study. Nevertheless, no theory has proven KD causality; however, the recurrent seasonal patterns continue to provide evidence supporting the association to hypothesized causal triggers and that the susceptibility of KD is multifactorial (2).

Although the presentation of KD normally occurs in young children under the age of 5 across all racial groups, the younger age presentation in our Black children when compared to White children is worth consideration (1). The gender distribution and young median age at KD hospitalization among Black children found in our study is similar to the overall median age for hospitalization and gender distribution reported by Bronstein et al. (4). Although the median age is not clinically low among Black children, it does differ from their White counterparts and is an important finding. Presenting with KD at a younger age has been associated with higher rates of coronary artery aneurysm (CAA) development despite timely diagnosis and treatment with IVIG (37). We have previously reported higher rates of CAA among Black children with KD (14). It is thought that certain children have higher susceptibility and may be triggered at a lower threshold, and therefore a younger age, whereas those that are less susceptible require a higher exposure threshold contributing to later presentation (13, 36). Moreover, sustained cases most of the year have also been observed among younger KD patients in Japan, similar to the case trend and age seen among Black children in our study (24, 38). Further exploration may be warranted for factors that drive the younger age presentation observed in Black patients compared to their White counterparts and also to examine how earlier age at presentation could have an association with poorer clinical outcomes in this group.

Our study holds several limitations. It is a single center study and therefore lacks generalizability for cases outside our geographical area or other sites; however the study site is the largest children's hospital and major referral institution in the State. Our study only captured children with KD who were hospitalized at our center and may have inadvertently excluded those treated elsewhere and/or cases who were not hospitalized. The small number of cases in the time series analysis did not allow for statistical comparison and could be biased. Also the incidence of KD mapped by county may not be representative as incidence may be influenced by sample size of both KD cases and county populations.

## Conclusion

KD incidence among White children in a Southern U.S. State follows the previously described seasonality in other U.S. regions with rising cases during winter and spring. However, the seasonality of KD among Black children differed from that of White children. In addition to rising cases in the winter and spring, high peaks similar to those observed in the winter were also seen in the summer and fall. The trend of cases observed among Black children with KD was similar to that of Japanese children a group with high susceptibility for KD. Further studies are needed to investigate inflammatory and environmental triggers for the increased susceptibility to develop KD among Black children when compared to other races.

## Data Availability

The datasets presented in this article are not readily available because data belongs to our institution. Requests to access the datasets should be directed to lpadilla@uabmc.edu.

## Supplementary material

Supplemental Figure 1A Geographical distribution by county of incident Kawasaki Disease hospitalizations among Black children.

Supplemental Figure 1B Geographical distribution by county of incident Kawasaki Disease hospitalizations among White children.
