# Supplementary figures and images for: Seasonality and geographical distribution of Kawasaki disease among Black children in the Southeast United States
Source: Front Pediatr. 2023 Jun 27;11:1203431. doi: 10.3389/fped.2023.1203431 (PMC10333540; doi:10.3389/fped.2023.1203431)

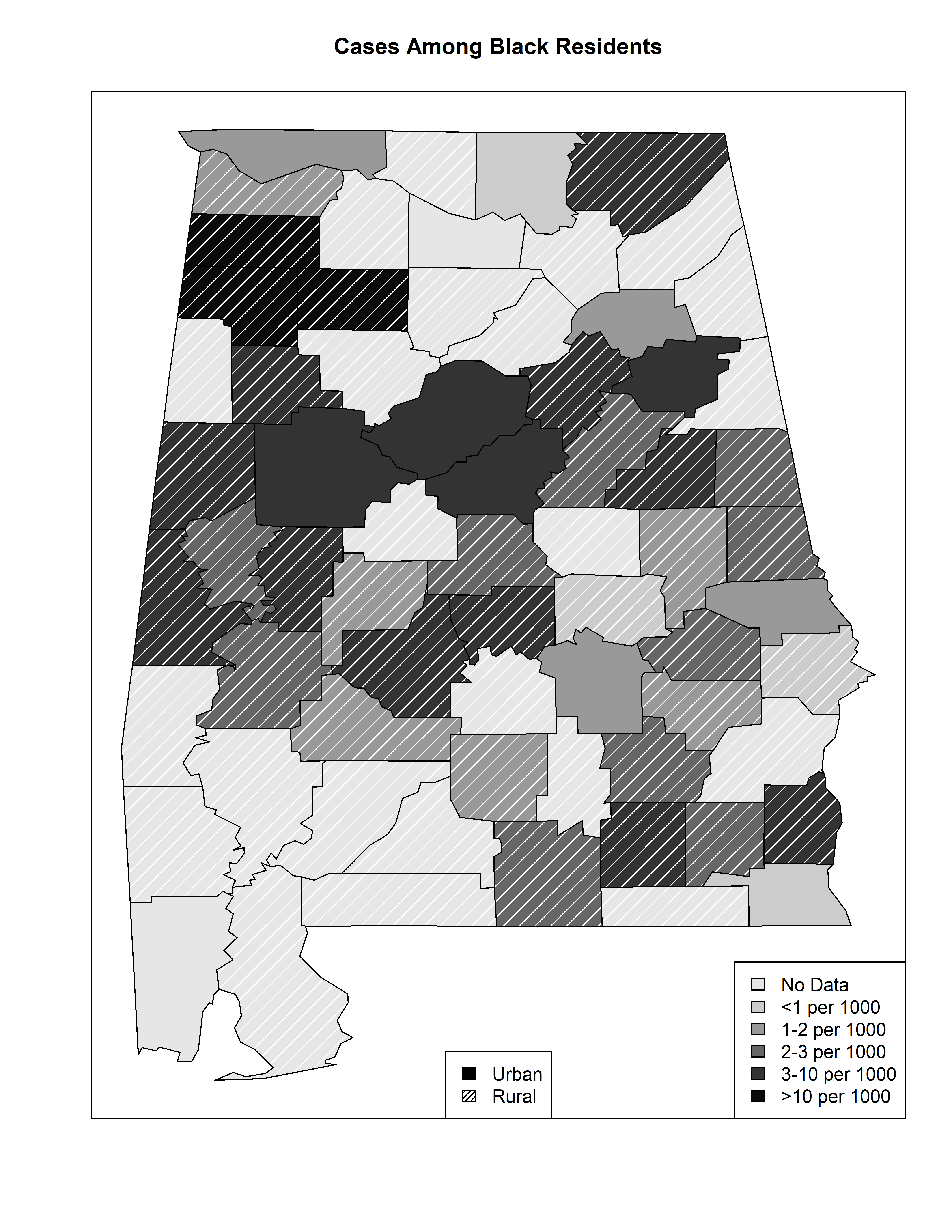

Supplement: Supplemental Figure 1A — Geographical distribution by county of incident Kawasaki Disease hospitalizations among Black children. [file Image1.tif]

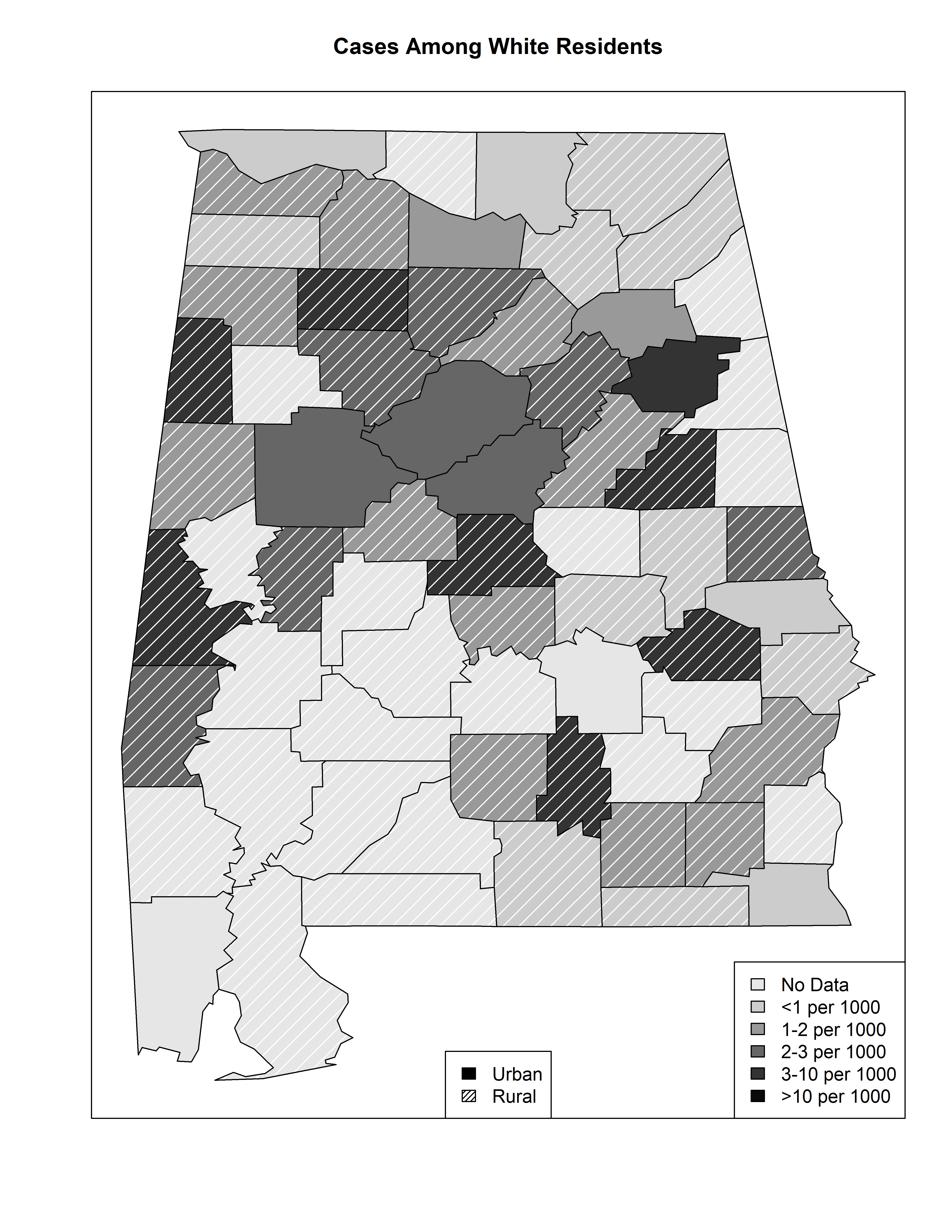

Supplement: Supplemental Figure 1B — Geographical distribution by county of incident Kawasaki Disease hospitalizations among White children. [file Image2.tif]
